# *MMP2* and* MMP10* Polymorphisms Are Related to Steroid-Induced Osteonecrosis of the Femoral Head among Chinese Han Population

**DOI:** 10.1155/2019/8298193

**Published:** 2019-05-05

**Authors:** Ye Tian, Feimeng An, Jiaqi Wang, Chang Liu, Huiqiang Wu, Yuju Cao, Jianzhong Wang, Guoqiang Wang

**Affiliations:** ^1^Inner Mongolia Medical University, Hohhot, Inner Mongolia, China; ^2^Department of Trauma Orthopedics, The Second Affiliated Hospital of Inner Mongolia Medical University, Hohhot, Inner Mongolia, China; ^3^Inner Mongolia Autonomous Region Hospital of Traditional Chinese Medicine, Hohhot, Inner Mongolia, China; ^4^Zhengzhou Traditional Chinese Medicine (TCM) Traumatology Hospital, Zhengzhou, Henan Province, China

## Abstract

**Background:**

Steroid-induced osteonecrosis of the femoral head is a relatively serious condition which seriously reduces patient quality of life. However, the pathogenesis of steroid-induced ONFH is still unclear. In recent years, more scholars have found that the pathogenesis of steroid-induced ONFH is related to susceptibility factors such as* MMPs*/*TIMPs *system. The main purpose of this study is to investigate the correlation between* MMP2 *and* MMP10* gene polymorphisms and steroid-induced ONFH in Chinese Han population.

**Methods:**

Six SNPs in* MMP2* and two SNPs in* MMP10* were genotyped using Agena MassARRAY RS1000 system from 286 patients of steroid-induced ONFH and in 309 healthy controls. The association between* MMP2* and* MMP10* polymorphisms and steroid-induced ONFH risk were estimated by the Chi-squared test, genetic model analysis, haplotype analysis, and stratification analysis. The relative risk was estimated by odd ratios (ORs) and 95% confidence intervals (CIs).

**Result:**

We found that the minor TG allele of rs470154 in* MMP10* was associated with an increased risk of steroid-induced ONFH (OR = 1.45, 95% CI, 1.03 – 2.05,* p *= 0.032). In the genetic model analysis, we found that rs2241146 in* MMP2* gene and rs470154 in* MMP10 *gene showed a statistically significant association with increased risk of steroid-induced ONFH. The six SNPs (rs470154, rs243866, rs243864, rs865094, rs11646643, and rs2241146) showed a statistically significant association with different clinical phenotypes.

**Conclusion:**

Our results verify that genetic polymorphisms of* MMP2* and* MMP10* contribute to steroid-induced ONFH susceptibility in the population of Chinese Han population, and our study provides new insights into the role that* MMP2* and* MMP10* plays in the mechanism of ONFH.

## 1. Introduction

Osteonecrosis of the femoral head (ONFH) is a debilitating bone disease, characterized by collapse of the femoral head and subsequent loss of hip joint function [1, 2]. ONFH frequently occurs in young and middle-aged people and seriously affects the quality of life for patients. At present, the number of new patients diagnosed with ONFH each year is 150,000 to 200,000 in China [3]. Widespread use of corticosteroids is widely believed to be one of the common causes of nontraumatic osteonecrosis. Steroid-induced ONFH accounts for 24.1% of all femoral head necrosis [4]. In clinical practice, we can often find that not all patients with a few months of steroid administration will develop steroid-induced ONFH, suggesting that genetic factors may confer susceptibility or resistance to steroid-induced ONFH. The certain genetic background has been shown related to ONFH in previous studies; single nucleotide polymorphism (SNP) is believed to be positively correlated with steroid-induced ONFH risk [5, 6].

Matrix metalloproteinases* (MMPs*), an enzyme family that is involved in the degradation of various proteins in the extracellular matrix (ECM), play an important part in the tissue remodeling process and physiological and pathological repair [7–9]. A complete set of* MMPs* produced by human cells is defined based on the complete human genome sequence. Recent genomic studies have shown that the* MMP* family has 24 distinct genetically encoded members, including* MMP2* and* MMP10 *[10]*. MMP2*, encoded by* MMP2 *gene in chromosome 16q12.2, is a 72-kDa type IV collagenase.* MMP2* is a rich enzyme secreted by osteoblasts and there is a significant negative correlation between* MMP2* levels and bone mineral density [11]. Keiichi Inoue's previous experiments demonstrated that* MMP2* is important to the progress of forming and maintaining the osteocyte canalicular network and its formation is a determinant of bone remodeling and mineralization [12, 13]. In addition, studies have shown that osteolysis can be caused by abnormal expression of* MMP2 *[14]. In the course of ONFH, articular cartilage degenerates in the early stages of ONFH before significant radiological changes occur in the femoral head; ONFH articular cartilage degradation indicators include* MMP2* [15].* MMP2* activates various cytokines and chemokines in inflammatory response [16]. Another gene* MMP10*, encoded by* MMP10* gene in chromosome 11q22.2, is a secreted metalloproteinase involved in the physiological process of bone growth and wound healing [17]. In the occurrence of periodontitis,* MMP10* may increase the risk of periodontitis; its mechanism is consistent with the local inflammatory response [18].

Studies have shown that the lack of functional* MMP10* may be involved in the calcification process in vivo and in vitro VSMC, leading to a decrease in the degree of calcification. This result was consistent with reports of impaired atherosclerotic calcification in* MMP2*-deficient mice [17]. In addition, Duits et al. demonstrated higher plasma* MMP2* and CSF* MMP10* levels in AD patients compared to VaD patients and controls [19]. In previous study, investigator found the association between genetic polymorphisms of* MMP2* and the risk of alcohol-induced osteonecrosis of the femoral head in the population of China [20]. Although* MMP2 *has been studied more extensively than* MMP10*, CSF* MMP10*, also known as lysostromin-2, has been linked to inflammation. However, there are no reports on the association between genetic polymorphisms of* MMP2 *and* MMP10 *and the risk of steroid-induced ONFH. Given the crucial role of* MMP2 *and* MMP10* in the inflammatory response, bone metabolism, and bone loss, we speculated that the polymorphism of* MMP2 *and* MMP10* might be associated with the risk of steroid-induced ONFH. Thus, we undertook the case-control study included 286 patients with steroid-induced ONFH and 309 healthy controls to elucidate the role of* MMP2* and* MMP10 *gene polymorphisms in the occurrence of steroid-induced ONFH among Chinese Han population.

## 2. Materials and Methods

### 2.1. Study Participants

From September 2014 to January 2016, a total of 595 subjects included 286 patients with steroid-induced ONFH and 309 healthy controls were consecutively recruited from Zhengzhou Traditional Chinese Medicine (TCM) Traumatology Hospital. The controls were enrolled based on a physical examination, with no steroid-induced ONFH or other related diseases. All subjects were Han people living in or near Zhengzhou City.

The inclusion criteria of cases were as follows: ONFH was diagnosed based on MRI and CT scanning. Steroid-induced osteonecrosis was diagnosed based on a history of taking a mean daily dose of 16.6 mg or an equivalent maximum daily dose of 80 mg of prednisolone within 1 year [21]. The exclusion criteria of cases were as follows: patients did not meet the diagnostic criteria of steroid-induced ONFH; patients with traumatic ONFH, dislocation of the hip joint, or other hip diseases were excluded; patients drank more than 400 ml per week or had significant familial hereditary disease.

This study complied with the principles of the Declaration of Helsinki. The Ethical Committee of Zhengzhou Traditional Chinese Medicine Traumatology Hospital agreed to the study and all participants provided written informed consent.

### 2.2. SNPs Selection and Genotyping

Based on data from the database (dbSNP and GWASs), eight SNPs of* MMP2* and* MMP10* were selected. All SNPs we selected had minor allele frequencies > 5% in the global population from the 1000 Genome HapMap database. We collected blood samples into EDTA tubes after centrifugation at 2000 rpm for 10 minutes. Blood samples kept at -80°C for future experiments. Genomic DNA was obtained from whole blood using GoldMag extraction method (GoldMag, China) and then stored at -20°C. We evaluated DNA quantity using spectrophotometry (DU530UV/VIS spectrophotometer, Beckman Instruments, Fullerton, CA, USA). Agena MassARRAY Assay Design 4.0 Software was implemented to develop the Multiplexed SNP Mass EXTEND assay. Agena MassARRAY RS1000 system was carried out to measure the SNP genotypes by the manufacturer's protocol. Data management and analyses were performed using the Agena Bioscience Typer 4.0 software.

### 2.3. Statistical Analyses

Statistical analyses were performed using the SPSS 20.0 software (SPSS, Chicago, IL). The Chi-square test was used to check whether the genotype distribution matched Hardy–Weinberg equilibrium (HWE) in the control group. In this study, all the tests were two-tailed, and* p* values less than 0.05 were regarded as statistically significant. We used Chi-squared test to calculate the distribution differences of allele, genotype, and haplotype. We analyzed associations between the genotypes of* MMP2* and* MMP10* polymorphisms and the risk of steroid-induced ONFH through different genetic models, codominant, dominant, recessive, and log-additive models by computing odds ratios (ORs) and 95% confidence intervals (CIs) from logistic regression adjusted for age and gender. The stratification analysis was performed with clinical stages, hip lesions, and course condition. Finally, haploview software (version 4.2) was used to estimate the linkage disequilibrium (LD) and haplotype construction.

## 3. Result

A total of 286 patients with steroid-induced ONFH (mean age: 41.83 ± 13.12) and 309 healthy controls (mean age: 48.78 ± 8.30) were recruited for our study. Basic characteristics, including gender, age, and the clinical phenotypes (unilateral or bilateral hip lesions, >12 months or ≤12 months course, and II, III, IV clinical stages of steroid-induced ONFH) were listed in Table 1. Steroid-induced ONFH cases and healthy controls were matched in sex (*p *= 0.412). However, we found the cases and healthy controls were not matched for age (*p *< 0.05), so we adjusted the age factor in the subsequent analysis.

Table 2 summarizes basic information about the candidate in the eight SNPs. None of the SNPs that were excluded deviated from Hardy–Weinberg equilibrium (HWE). The *χ*^2^ test was utilized to calculate the differences in frequency distribution of alleles between cases and controls. We found that rs470154 in* MMP10* was linked to an increased risk of steroid-induced ONFH (OR = 1.45, 95% CI: 1.03 –2.05,* p *= 0.035).

Subsequently, we evaluated the association between these SNPs and steroid-induced ONFH risk using four genetic models (codominant, dominant, recessive, and log-additive) by logistic-regression analysis.  Table 3 showed that rs2241146 in the* MMP2* gene was associated with an increased risk of steroid-induced ONFH in the recessive model before (OR = 2.15, 95% CI: 1.05–4.41,* p *= 0.032) and after (adjusted OR = 2.42, 95% CI: 1.14–5.14,* p *= 0.018) adjustment. Moreover, rs470154 in* MMP10 *polymorphism was correlated with an increased risk of steroid-induced ONFH in the log-additive model before adjustment for age and gender (OR = 1.44, 95% CI: 1.02–2.02,* p* = 0.035).

Next, cases were stratified in terms of unilateral or bilateral hip lesions, > 12 months or ≤ 12 months course, and clinical stages. We further evaluated the effects of alleles and genotypes of SNPs on the risk of steroid-induced ONFH among the different subgroups (Table 4). In the allele and genotype models, the locus associated with steroid-induced risk of ONFH in patients with unilateral femoral head necrosis was rs243866 (adjusted OR = 0.40, 95% CI: 0.20–0.80,* p *= 0.007;* p *= 0.013), rs243864 (adjusted OR = 0.40, 95% CI: 0.20–0.78,* p *= 0.006;* p *= 0.010), and rs865094 (adjusted OR = 1.56, 95% CI: 1.08–2.24,* p *= 0.016;* p *= 0.029). We found that rs11646643 and rs2241146 were only associated with steroid-induced ONFH risk in patients with unilateral femoral head necrosis in the allele model. Among them, rs2241146 was associated with steroid-induced femoral head necrosis in the genotype model (unilateral,* p *= 0.038; aged > 12 months,* p *= 0.013). Moreover, in the allele model, rs470154 was associated with steroid-induced ONFH risk in patients with bilateral femoral head necrosis (adjusted OR = 1.50, 95% CI: 1.04–2.18,* p *= 0.031), clinical stage III (adjusted OR = 1.76, 95% CI: 1.17–2.67;* p *= 0.007), ≤ 12 months course (adjusted OR = 1.71, 95% CI: 1.13–2.59,* p *= 0.010). In the genotype model, it was related to clinical stage III (*p *= 0.015). In general, the SNPs with protective factors were rs243866, rs243864, and rs11646643. Separately, the SNPs with increase risk were rs470154, 865094, and rs2241146. In the case-control study, we performed a power analysis of selected* MMP2* and* MMP10* SNPs (Table 5).

**Figure 1:**
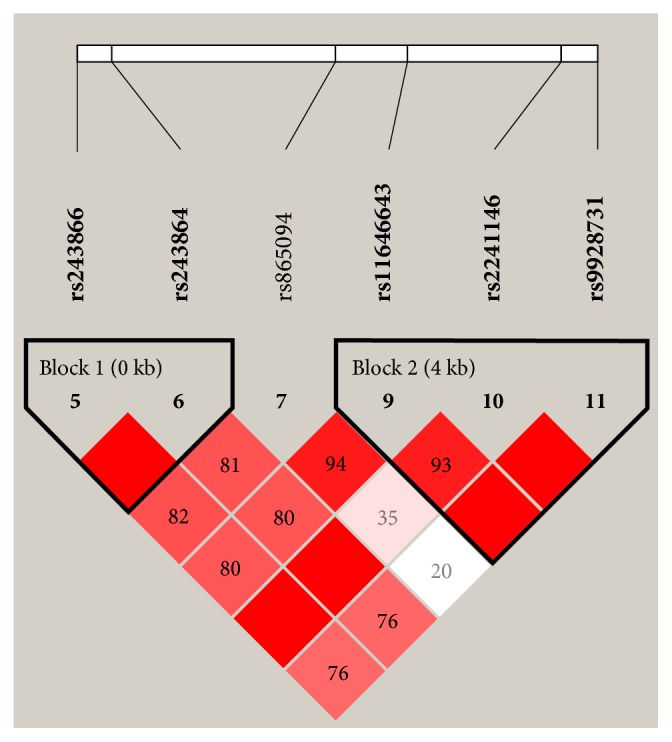
Linkage disequilibrium (LD) plots containing six SNPs from* MMP2*. Red squares display statistically significant associations between a pair of SNPs, as measured by D'; darker shades of red indicate higher D'.

Finally, the parameter D′ was used to analyze the extent of linkage disequilibrium between SNPs. Then the haplotype LD block was determined according to the control group data (Figure 1). Two blocks were detected by haplotype analysis in the* MMP2* gene (Figure 1), one block comprising rs243866 and rs243864 and the other one comprising rs11646643, rs2241146, and rs9928731. However, we did not find any association between the haplotype in the* MMP2* and steroid-induced ONFH risk (Tables 6 and 7).

## 4. Discussion

In this study, we found rs2241146 and rs470154 were significantly associated with an increased risk of steroid-induced ONFH. Six SNPs showed a statistically significant association with different clinical phenotypes (rs470154, rs243866, rs243864, rs865094, rs11646643, and rs2241146). To our knowledge, this is the first report to investigate an association between these loci and steroid-induced ONFH susceptibility.

*MMP2* has been taken into account as a target candidate gene of genetic association studies for numerous human diseases. Robert Wojciechowski et al. [22] study showed that rs9928731 in* MMP2 *was significantly associated with ocular refraction in the AMISH families, which implicated* MMP2* as regulator of nonpathologic refractive error. Ou Liu et al. [23] found that the* MMP2* haplotype rs2241145-rs9928731 showed the significant association with Thoracic Aortic dissection (TAD), which may indicate the genetic predisposition to TAD in Chinese Han population. Rs9928731, rs11646643, and rs2241146 have a strong linkage between each pair of the three SNPs. In our study, however, there was no association between rs9928731 and steroid-induced ONFH. Inconsistent data observed between single-label analysis and haplotype analysis may indicate that rs9928731 was not independently associated with ONFH risk, but rather the SNPs in combination. Rs9928731 is located on chr16 of the* MMP2 *intron region between the sixth and seventh exons in* MMP2*, it does not represent a coding variant and located near the region where acetylation of histone H3 lysine 27 is significantly enriched, which is a reliable chromatin marker of active enhancers. Noncoding variants in these regions can result in damage to regulatory functions, and in some cases are supposed to cause disease phenotypes. In addition, epigenetic regulation of gene expression by histone modifications is cell-type specific. In the relevant region, H3 lysine 27 acetylation is highly enriched in human fibroblasts (*MMP2 *region) and human keratinocytes (*MMP1 *through* MMP10* intergenic region); SNPs are also possible [22, 24].

Juiz et al. [25] indicated that rs243866 may be involved in the ECM remodeling. Additionally, previous studies showed that A allele carriers may be related to decreased risk of systolic heart failure in a Han Chinese population [26].* MMP2 *rs243866 is located in 5 to a half palindromic potential estrogen receptor binding site. The 1575G allele acts as an enhancer in estrogen receptor-positive MCF-7 cells, while the 1575A allele reduces transcriptional activity. Gel migration assays confirmed that the binding of the estrogen receptor to this region was affected by differences in allelic expression [27]. Located in the GATA-1 site (CTATCT) of the promoter region of* MMP2*, the rs243866 polymorphism has binding sites for transcription factors AP-2, p53, Sp1, and Sp3. Protease expression is regulated by these transcription factors by the transcription rate of the gene [28]. For rs243864 polymorphism, a paper reported that the rs243864, rs243865, 243866 haplotype was statistically significant related to childhood obesity [29]. In addition, rs243864 is present in the gene promoter, which is a tag SNP of the rs243865 and rs243866 polymorphisms [30]. It has been reported that genetic associations in the* MMP2* promoter may be caused by changes in* MMP *expression, because the three SNPs studied are functional SNPs that affect transcriptional activity.

Our study also found that* MMP2 *gene rs243866 and rs243864 may be the two protective SNPs of steroid-induced ONFH. The strong linkage between each pair of two SNPs (rs243866 and rs243864) confirmed that rs243866 and rs243864 could act not only alone but also coinitiate disease. In the Mexican mestizo population, rs243864 and rs11646643 of the* MMP2* gene were associated with the high risk of COPD [28]. Our conclusion was that rs11646643 may be a low-risk factor of unilateral steroid-induced ONFH. And there was a strong linkage between each pair of the three SNPs (rs11646643, rs2241146, and rs9928731). But we did not find an association between steroid-induced ONFH and the above haplotype. In the study of Alicia Beeghly-Fadiel [31], rs2241146 may be unrelated to breast cancer risk. In this study, we found rs2241146 may be a high-risk factor of steroid-induced ONFH (especially unilateral ONFH).

Similarly, in our study, we found that rs470154 SNP in* MMP10* gene had an increased risk steroid-induced of ONFH (especially bilateral ONFH, clinical stage III, and ≤ 12 months course). The other study showed that there is no association between rs470154 and the susceptibility to disc degeneration [32]. Information on specific genetic variants is inconsistent across different diseases, which can be explained by genetic differences between these diseases. It also illustrates the complexity of the mechanism involved in promoting SNP in ONFH. The differences observed here do not necessarily contradict previous reports. Further research is needed in populations with different ethnic and genetic backgrounds.

There were some important discoveries revealed in our study, but some limitations of this study should be considered when interpreting these results. First of all, our study did not include an analysis of biological functions, which will be crucial for elucidating the role of the gene in steroid-induced ONFH. Secondly, the sample size was relatively small, which might convert the positive findings into negative results. A larger case-control study is expected to circumvent those problems, which could make our conclusions more powerful. Although we obtained evidence for the association between* MMP2* and* MMP10* gene polymorphisms and steroid-induced ONFH risk, the pathogenesis of steroid-induced ONFH was still not completely understood. Further studies should be carried out to verify the present study in other ethnicity.

## 5. Conclusions

The* MMP10* may be related to increased risk of steroid-induced ONFH. It is the first time that our results reveal an association between a* MMP2 *SNP at the rs2241146 locus and an increased risk of steroid-induced ONFH in Chinese Han population. And we found statistically significant differences in clinical phenotypes among the six SNPs (rs470154, rs243866, rs243864, rs865094, rs11646643, and rs2241146). From the genetic point of view to explore the pathogenesis of steroid-induced ONFH, early detection of susceptible population, timely intervention, and active prevention, the current treatment model can be transferred from postoperative surgery to predisease prevention. This study provides an important data source for the establishment of a bone necrosis gene bank in China. It can be used as a molecular marker for the diagnosis of osteonecrosis in the future, which not only benefits the majority of patients, but also has significant economic and social significance.

## Figures and Tables

**Table 1:** Characteristics of the individuals in controls and steroid-induced ONFH patients.

Variables	Cases (n=286)	Controls (n=309)	*p* value
	N (%)	N (%)	
Age, years			
Mean ± SD	41.83 ± 13.12	48.78 ± 8.30	< 0.001^a^
≤48	203 (71.0)	170 (55.0)	
>48	83 (29.0)	139 (45.0)	
Gender			0.412^b^
female	113 (39.5)	112 (36.2)	
male	173 (60.5)	197 (63.8)	
Clinical stages			
Stage II	64 (22.4)		
Stage III	126 (44.1)		
Stage IV	96 (33.6)		
Hip lesions			
Unilateral	83 (29.0)		
Bilateral	203 (71.0)		
Course, months			
>12	157 (45.1)		
≤12	129 (54.9)		

*P* < 0.05 indicates statistical significance.

^a^ Independent samples t test.

^b^ Two-sided Chi-squared test.

**Table 2:** Basic information of candidate SNPs in this study.

SNP	Gene	Position	Alleles	MAF		*p* ^a^ value	ORs	95% CI		*p* ^b^ value
			A/B	case	control	for HWE				
rs243866	*MMP2*	16q12.2	A/G	0.112	0.173	0.329	0.79	0.56	1.13	0.194
rs243864	*MMP2*	16q12.2	G/T	0.112	0.140	0.342	0.78	0.55	1.10	0.151
rs865094	*MMP2*	16q12.2	G/A	0.290	0.266	1.000	1.13	0.87	1.45	0.357
rs11646643	*MMP2*	16q12.2	G/A	0.175	0.205	0.601	0.83	0.62	1.11	0.202
rs2241146	*MMP2*	16q12.2	A/G	0.234	0.213	0.611	1.13	0.86	1.48	0.388
rs9928731	*MMP2*	16q12.2	T/C	0.486	0.476	0.170	1.04	0.83	1.31	0.719
rs470154	*MMP10*	11q22.2	T/G	0.149	0.107	1.000	1.45	1.03	2.05	0.032∗
rs17293607	*MMP10*	11q22.2	G/A	0.003	0.005	1.000	0.72	0.12	4.32	0.931

SNP: single nucleotide polymorphism, HWE: Hardy-Weinberg equilibrium, OR: odds ratio, 95% CI: 95% confidence interval, and MAF: minor allele frequency.

^a^ *P *values were calculated by exact test.

^b^ *P* values were calculated by Pearson Chi-squared test.

**Table 3:** Genotypic model analysis of relationship between SNPs and steroid-induced ONFH.

SNP ID	Model	Genotype	Controls	Cases	Without Adjustment		With Adjustment	
					OR (95% CI)	*p *value	OR (95% CI)	*p * ^*a*^ value
rs470154	Codominant	G/G	245 (79.5%)	210 (73.4%)	1	0.067	1	0.120
		G/T	60 (19.5%)	67 (23.4%)	1.30 (0.88-1.93)		1.27 (0.84-1.92)	
		T/T	3 (1%)	9 (3.1%)	3.50 (0.94-13.10)		3.37 (0.83-13.67)	
	Dominant	G/G	245 (79.5%)	210 (73.4%)	1	0.078	1	0.130
		G/T-T/T	63 (20.4%)	76 (26.6%)	1.41 (0.96-2.06)		1.36 (0.91-2.04)	
	Recessive	G/G-G/T	305 (99%)	277 (96.8%)	1	0.055	1	0.083
		T/T	3 (1%)	9 (3.1%)	3.30 (0.89-12.33)		3.20 (0.79-12.95)	
	Log-additive	-* *-* *-	-* *-* *-	-* *-* *-	1.44 (1.02-2.02)	0.035*∗*	1.40 (0.98-2.00)	0.066
rs2241146	Codominant	G/G	188 (61.2%)	175 (61.2%)	1	0.078	1	0.058
		G/A	107 (34.9%)	88 (30.8%)	0.88 (0.62-1.25)		0.93 (0.65-1.34)	
		A/A	12 (3.9%)	23 (8%)	2.06 (0.99-4.26)		2.36 (1.10-5.07)	
	Dominant	G/G	188 (61.2%)	175 (61.2%)	1	0.990	1	0.710
		G/A-A/A	119 (38.8%)	111 (38.8%)	1.00 (0.72-1.39)		1.07 (0.75-1.51)	
	Recessive	G/G-G/A	295 (96.1%)	263 (92%)	1	0.032*∗*	1	0.018*∗*
		A/A	12 (3.9%)	23 (8%)	2.15 (1.05-4.41)		2.42 (1.14-5.14)	
	Log-additive	-* *-* *-	-* *-* *-	-* *-* *-	1.12 (0.86-1.46)	0.400	1.19 (0.90-1.58)	0.230

^a^ *P* values were calculated by Wald test by unconditional logistic regression adjusted for age and gender.

*∗ P* < 0.05 indicates statistical significance.

**Table 4:** Stratified analysis for association of *MMP2* and* MMP10* gene polymorphism with the clinical phenotypes of steroid-induced ONFH.

SNPs	Subgroups	Genotype(n)		Allele(%)			
		AA/AB/BB	*p* ^a^	OR	95% CI		*p* ^b^
rs470154(T/G)	Controls	3 / 60 / 245					
	Case-Unilateral	4 / 15 / 64	0.094	1.34	0.81	2.23	0.258
	Case-Bilateral	5 / 52 / 146	0.250	1.50	1.04	2.18	0.031*∗*
	Case-Stage II	3 / 11 / 50	0.130	1.28	0.72	2.26	0.401
	Case-Stage III	5 / 34 / 87	0.015*∗*	1.76	1.17	2.67	0.007*∗*
	Case-Stage IV	1 / 22 / 73	0.820	1.19	0.72	1.96	0.492
	Case > 12 months	5 / 31 / 121	0.190	1.25	0.83	1.90	0.290
	Case ≤ 12 months	4 / 36 / 89	0.074	1.71	1.13	2.59	0.010*∗*
rs243866 (A/G)	Controls	8 / 68 / 231					
	Case-Unilateral	0 / 10 / 73	0.013*∗*	0.40	0.20	0.80	0.007*∗*
	Case-Bilateral	3 / 48 / 152	0.470	0.97	0.67	1.40	0.862
	Case-Stage II	1 / 10 / 53	0.400	0.65	0.35	1.23	0.187
	Case-Stage III	1 / 21 / 104	0.240	0.63	0.39	1.03	0.064
	Case-Stage IV	1 / 27 / 68	0.240	1.12	0.71	1.77	0.620
	Case > 12 months	2 / 32 / 123	0.470	0.82	0.54	1.24	0.341
	Case ≤ 12 months	1 / 26 / 102	0.240	0.77	0.49	1.21	0.255
rs243864 (G/T)	Controls	8 / 70 / 230					
	Case-Unilateral	0 / 10 / 73	0.010*∗*	0.40	0.20	0.78	0.006*∗*
	Case-Bilateral	3 / 48 / 152	0.500	0.95	0.66	1.36	0.764
	Case-Stage II	1 / 10 / 53	0.360	0.64	0.34	1.20	0.163
	Case-Stage III	1 / 21 / 104	0.200	0.62	0.38	1.01	0.051
	Case-Stage IV	1 / 27 / 68	0.270	1.10	0.69	1.73	0.692
	Case > 12 months	2 / 32 / 123	0.470	0.80	0.53	1.21	0.286
	Case ≤ 12 months	1 / 26 / 102	0.230	0.75	0.48	1.18	0.213
rs865094 (G/A)	Controls	22 / 120 / 166					
	Case-Unilateral	8 / 44 / 31	0.029*∗*	1.56	1.08	2.24	0.016*∗*
	Case-Bilateral	9 / 88 /106	0.430	0.97	0.73	1.29	0.855
	Case-Stage II	4 / 30 / 30	0.500	1.16	0.77	1.77	0.478
	Case-Stage III	9 / 58 / 59	0.780	1.19	0.86	1.64	0.290
	Case-Stage IV	4 / 92 / 48	0.370	1.02	0.71	1.47	0.900
	Case > 12 months	9 / 70 / 78	0.750	1.07	0.79	1.45	0.649
	Case ≤ 12 months	8 / 62 / 59	0.240	1.19	0.87	1.65	0.277
rs11646643 (G/A)	Controls	11 / 104 / 193					
	Case-Unilateral	1 / 18 / 64	0.062	0.53	0.32	0.88	0.014*∗*
	Case-Bilateral	5 / 70 /127	0.320	0.96	0.70	1.31	0.800
	Case-Stage II	2 / 22 / 40	0.640	0.99	0.62	1.59	0.971
	Case-Stage III	2 / 38 / 86	0.360	0.78	0.53	1.14	0.200
	Case-Stage IV	2 / 28 / 65	0.430	0.79	0.51	1.21	0.273
	Case > 12 months	4 / 44 / 108	0.370	0.78	0.54	1.11	0.166
	Case ≤ 12 months	2 / 44 / 83	0.320	0.89	0.61	1.29	0.532
rs2241146 (A/G)	Controls	12 / 107 / 188					
	Case-Unilateral	10 / 30 / 43	0.038*∗*	1.59	1.08	2.33	0.017*∗*
	Case-Bilateral	13 / 58 / 132	0.230	0.96	0.71	1.31	0.804
	Case-Stage II	6 / 14 / 44	0.076	0.94	0.59	1.51	0.797
	Case-Stage III	7 / 44 / 75	0.650	1.10	0.78	1.57	0.587
	Case-Stage IV	10 / 30 / 56	0.078	1.30	0.89	1.89	0.173
	Case > 12 months	17 / 51 / 89	0.013*∗*	1.37	1.00	1.88	0.050
	Case ≤ 12 months	6 / 37 / 86	0.710	0.86	0.60	1.25	0.435
rs9928731 (T/C)	Controls	63 / 166 / 78					
	Case-Unilateral	17 / 43 / 23	0.970	0.95	0.68	1.35	0.789
	Case-Bilateral	47 / 107 / 49	0.860	1.08	0.84	1.39	0.542
	Case-Stage II	15 / 29 / 20	0.550	0.94	0.64	1.38	0.763
	Case-Stage III	28 / 72 / 26	0.600	1.14	0.85	1.53	0.387
	Case-Stage IV	21 / 49 / 26	0.980	0.99	0.72	1.37	0.969
	Case > 12 months	27 / 88 / 42	0.670	0.91	0.69	1.20	0.500
	Case ≤ 12 months	37 / 62 / 30	0.270	1.23	0.92	1.64	0.164

*∗P*<0.05 indicates statistical significance.

^a^ *P* values were calculated by Wald test adjusted for age and gender.

^b^ *P* values were calculated by Pearson Chi-squared test.

**Table 5:** A power analysis of selected SNPs for *MMP2* and *MMP10* in case-control studies.

SNP_ID	Case n_1_	Control n_2_	Case A	Control A	Case *p*_*1*_	Control *p*_*2*_	*p*	z_*β*_	power
rs243866	572	614	64	84	0.112	0.137	0.125	-0.665	0.253
rs243864	572	616	64	86	0.112	0.140	0.126	-0.525	0.300
rs865094	572	616	166	164	0.290	0.266	0.278	-1.037	0.150
rs11646643	570	616	100	126	0.175	0.205	0.191	-0.687	0.246
rs2241146	572	614	134	131	0.234	0.213	0.223	-1.095	0.137
rs9928731	572	614	278	292	0.486	0.476	0.481	-1.600	0.055
rs470154	572	616	85	66	0.149	0.107	0.127	0.183	0.573
rs17293607	572	618	2	3	0.003	0.005	0.004	-1.608	0.054

*p*<0.05 indicates statistical significance.

**Table 6:** The haplotype frequencies of *MMP2* polymorphisms and their association with steroid-induced ONFH risk.

Haplotype			Freq	OR^a^(95% CI)	*p* ^a^
rs11646643	rs2241146	rs9928731			
A	G	T	0.481	1	-
A	A	C	0.220	1.13 (0.84 - 1.54)	0.420
G	G	C	0.188	0.84 (0.60 - 1.19)	0.330
A	G	C	0.108	0.86 (0.57 - 1.29)	0.470

*p* < 0.05 indicates statistical significance.

a= adjusted by gender and age.

**Table 7:** The haplotype frequencies of* MMP2* polymorphisms and their association with steroid-induced ONFH risk.

Haplotype		Freq	OR^a^(95% CI)	*p* ^a^
rs243866	rs243864			
G	T	0.874	1	-
A	G	0.125	0.77 (0.53 - 1.10)	0.150

*p* < 0.05 indicates statistical significance.

a= adjusted by gender and age.

## Data Availability

The data used to support the findings of this study are available from the corresponding author upon request.
